# Incidence, risk factors, timing, and outcome of influenza versus COVID-19–associated putative invasive aspergillosis

**DOI:** 10.1017/ice.2020.460

**Published:** 2020-09-09

**Authors:** Camille Sarrazyn, Sofie Dhaese, Birgit Demey, Stefaan Vandecasteele, Marijke Reynders, Jens T. Van Praet

**Affiliations:** 1 Department of Nephrology and Infectious Diseases, AZ Sint-Jan Brugge-Oostende AV, Brugge, Belgium; 2 Faculty of Medicine and Health Sciences, Ghent University, Ghent, Belgium; 3 Department of Laboratory Medicine, Medical Microbiology, AZ Sint-Jan Brugge-Oostende AV, Brugge, Belgium; 4 Department of Internal Medicine and Pediatrics, Ghent University, Ghent, Belgium

*To the Editor*—Invasive pulmonary aspergillosis (IPA) is a well-established superinfection of influenza, especially in critically ill patients admitted to the intensive care unit (ICU).^1^ The incidence of this complication depends on the definition used and environmental factors, and classical host risk factors for IPA are generally absent in these patients. More recently, several reports have emerged of coronavirus disease 2019 (COVID-19)–associated IPA.^2–4^ However, whether the risk and morbidity of IPA is comparable between both viral illnesses remains unclear. We compared the incidence, risk factors, timing, and outcome of influenza and COVID-19–associated IPA for the 2019–2020 season in our institution, a 1,182-bed acute- and tertiary-care hospital in Belgium consisting of 3 separate campuses.

To study influenza-associated IPA, we included all consecutively admitted outpatients between October 1, 2019, and March 30, 2020, who met the World Health Organization (WHO) severe acute respiratory infection (SARI) case definition with a positive combined nasopharyngeal or throat swab for influenza RNA, tested by real-time (RT)-PCR on Taqman array cards (n = 141, median age, 66 years; interquartile range [IQR], 47–78; 47% female). Overall, 92 patients had influenza A H1N1; 33 had influenza A H3N2; 5 had influenza B; and 11 patients had influenza that could not be typed due to the low viral concentration.

For the analysis of COVID-19–associated IPA, we collected the data for all consecutively admitted outpatients between March 11, 2020, and April 17, 2020, with a positive nasopharyngeal swab (RT-PCR testing) for and/or IgG antibodies (MPIA on Architect-I System from Abbott, Sligo, Ireland) against severe acute respiratory coronavirus virus 2 (SARS-CoV-2) upon admission (n = 131; median age, 67 years; IQR, 56–79; 40% female). For the definition of IPA, we used ‘putative IPA’ because the optimal criteria for COVID-19–associated IPA have not been established.^5^ This definition requires the identification of *Aspergillus* spp in culture of bronchoalveolar lavage fluid or meeting at least 2 of the following conditions: the identification of *Aspergillus* spp in a culture of bronchial aspirate, positive galactomannan detection in bronchial aspirate or bronchoalveolar lavage fluid (Platelia antigen EIA from Bio-Rad, Berkeley, CA, with a cut-off index of > 1.00) or positive RT-PCR for *Aspergillus* spp in bronchial aspirate or bronchoalveolar lavage fluid. The latter 2 tests are routinely performed in our laboratory on all lower respiratory tract samples. For COVID-19 patients, the microbiological tests were performed on sputum or bronchial aspirate, which was routinely collected 3 times weekly from ventilated patients, given the risk of aerosolization during bronchoscopy.^6^ Patients were considered at risk for IPA the first 28 days after diagnosis of viral illness, or until death. Negative binomial regression was used to calculate the incidence and 95% confidence interval of IPA in patients with influenza or COVID-19.

Putative IPA was diagnosed in 5 patients of the influenza cohort (3.5%) and 4 of the COVID-19 cohort (3%), conferring to incidences of 218.7 per 100 patient years (95% CI, 40.1–1,627.9) and 118.8 per 100 patient years (95% CI, 21.6–834.2), respectively. All influenza types of the patients with influenza-associated IPA were A/H1N1. The clinical and microbiological characteristics of both patient groups are shown in Table 1. The time between admission and diagnosis of IPA was not different: a median of 6 days for influenza versus 4 days for COVID-19. All patients with COVID-19–associated IPA died, compared to 3 out of 5 influenza-associated IPA patients; all of these patients were ventilated in the ICU.

**Table 1.** The Clinical and Microbiological Characteristics of Patients With Influenza or COVID-19–Associated IPA During the 2019–2020 Season

Characteristic	Influenza Patients	COVID-19 Patients
Age, median y (range)	73 (66–79)	75 (67–89)
Sex, male	4/5	3/4
Positive galactomannan^a^	5/5	4/4
Positive PCR^a^	5/5	2/4 (2 tests ND)
Positive culture for *Aspergillus* spp^a^	3/5	4/4
Immunocompromised according due to EORTC/MSG criteria	2/5	0/4
Hematological malignancy	1/5	0/4
Neutropenia	2/5	0/4
COPD	4/5	0/4
Steroid use within 28 days before admission	2/5	0/4
BMI > 30	2/5	2/4
CKD stage III or higher	1/5	1/4
ICU admission	3/5	4/4
Mechanical ventilation	3/5	4/4
Antifungal treatment	Voriconazole and caspofungin (n=1), voriconazole (n=3), death before treatment initiation (n=1)	Voriconazole (n=1), voriconazole followed by liposomal amphotericin B (n=2), death before treatment initiation (n=1)

Note. IPA, invasive pulmonary aspergillosis; PCR, polymerase chain reaction assay; ND, not determined; EORTC/MSG, European Organization for Research and Treatment of Cancer/Mycoses Study Group; COPD, chronic obstructive pulmonary disease; BMI, body mass index, CKD, chonic kidney disease, ICU, intensive care unit.

a These tests were performed on lower respiratory tract samples.

In this single-center analysis, we found a similar incidence of IPA as superinfection of influenza and COVID-19. However, IPA was only observed in COVID-19 patients who needed mechanical ventilation, whereas IPA was also seen in influenza patients hospitalized outside the ICU. As described previously, most influenza and all COVID-19 patients lacked the classical host risk factors for IPA in our series. The timing of the IPA diagnosis was also comparable in both groups. We observed a very poor prognosis of both influenza and COVID-19–associated IPA in our ICU patients. Notably, galactomannan detection is not validated on non–bronchoalveolar lavage fluid respiratory samples, which might have led to overdiagnosis of putative IPA in COVID-19 patients. Nevertheless, our findings support the implementation of active surveillance for IPA early after admission in mechanically ventilated COVID-19 patients.
